# Structural equation model testing and the quality of natural killer cell activity measurements

**DOI:** 10.1186/1471-2288-5-1

**Published:** 2005-01-06

**Authors:** Leslie A Hayduk, Hannah Pazderka-Robinson, Greta G Cummings, Merry-Jo D Levers, Melanie A Beres

**Affiliations:** 1 Department of Sociology, University of Alberta, Edmonton, Alberta, T6G 2H4 Canada; 2 University Centre for Neuroscience, University of Alberta, Edmonton, Alberta, T6G 2S2 Canada; 3 Faculty of Nursing, University of Alberta, Edmonton, Alberta, T6G 2G3 Canada

## Abstract

**Background:**

Browne et al. [Browne, MacCallum, Kim, Andersen, Glaser: When fit indices and residuals are incompatible. Psychol Methods 2002] employed a structural equation model of measurements of target cell lysing by natural killer cells as an example purportedly demonstrating that small but statistically significant ill model fit can be dismissed as "negligible from a practical point of view".

**Methods:**

Reanalysis of the natural killer cell data reveals that the supposedly negligible ill fit obscured important, systematic, and substantial causal misspecifications.

**Results:**

A clean-fitting structural equation model indicates that measurements employing higher natural-killer-cell to target-cell ratios are more strongly influenced by a progressively intrusive factor, whether or not the natural killer cell activity is activated by recombinant interferon γ (rIFN γ). The progressive influence may reflect independent rate limiting steps in cell recognition and attachment, spatial competition for cell attachment points, or the simultaneous lysings of single target cells by multiple natural killer cells.

**Conclusions:**

If the progressively influential factor is ultimately identified as a mere procedural impediment, the substantive conclusion will be that measurements of natural killer cell activity made at lower effector to target ratios are more valid. Alternatively, if the individual variations in the progressively influential factor are modifiable, this may presage a new therapeutic route to enhancing natural killer cell activity. The methodological conclusion is that, when using structural equation models, researchers should attend to significant model ill fit even if the degree of covariance ill fit is small, because small covariance residuals do not imply that the underlying model misspecifications are correspondingly small or inconsequential.

## Background

Browne, MacCallum, Kim, Andersen and Glaser [1] employed a measurement model of natural killer cell lysis as an example of testing structural equation models. Their model failed to fit the data, though the authors judged the degree of covariance ill fit to be "negligible from a practical point of view"[1]. One of us (Hayduk) was engaged in a SEMNET [2] discussion of model fit testing, and objected to the close-yet-failing structural equation model being described as adequate. We re-examined the relevant measurement procedures and subsequently located a cleanly fitting model which provided evidence of important systematic effects coordinated with the effector to target ratios used during the measurement of natural killer (NK) cell activity. This article summarises the Browne et al. [1] data, discusses the clean-fitting model, and investigates alternative models in an attempt to better characterise the factor that produces the progressive measurement interference.

## Methods

### The immune system measurements

Browne et al. [1] analysed the correlation matrix for eight measures of immune system function of 72 females with breast cancer, recorded during investigation of the physiological consequences of a psychological intervention [3,4]. Four ^51^Cr-release measures of natural killer cell lysis were obtained using effector (NK cell) to target cell (K562 human myeloid cell) ratios of 100:1, 50:1, 25:1 and 12.5:1. Following Browne et al. [1] we designate these measures by their effector to target (E:T) ratios, NK100, NK50, NK25 and NK12 respectively. Similarly, natural killer cell lysis measured in the presence of recombinant interferon gamma (rIFNγ) using E:T ratios of 50:1, 25:1, 12.5:1 and 6.25:1, are designated IFN50, IFN25, IFN12, and IFN6 respectively. Lower E:T ratios are used in the presence of rIFNγ because rIFNγ increases NK cells' ability to rupture target cells.

**Figure 1:**
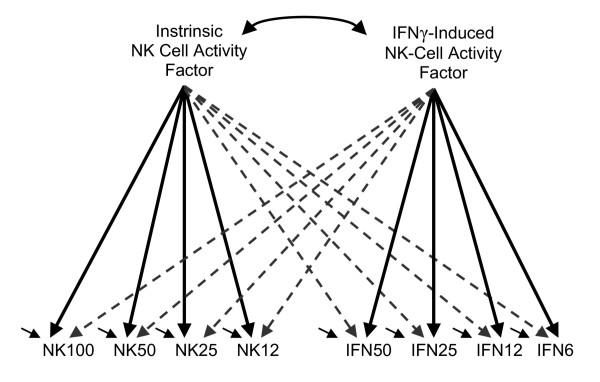
**The Browne, MacCallum, Kim, Andersen and Glaser (2002) two-factor model **The dashed arrows correspond to weak effects. Statistical identification of the model's coefficients requires exclusion of one dashed arrow from each factor as explained in Table 1.

The correlations reported in Browne et al.'s [1] Table 1 indicate that the four NK measures correlate highly with one another (average *r *= 0.852), and that the four rIFNγ enhanced NK measures also correlate highly with one another (averaging 0.960). However, the low correlations between the sets of NK and rIFNγ measurements (averaging only .111) indicate that the two sets of measurements reflect relatively distinct aspects of natural killer cell functioning. Browne et al. [1] viewed this as justifying the use of an exploratory two-factor model (Figure 1) which, unfortunately, was significantly inconsistent with the data (χ^2 ^= 103.59, degrees of freedom (*df*) = 13, and probability *p *< 10^-15^). The small but significant residual differences between the data correlations and the correlations implied by the two-factor model were dismissed by Browne et al.[1] as "negligible from a practical point of view". SEMNET discussion of this model prompted Hayduk to investigate whether some unrecognized measurement feature was producing the significant, even if seemingly slight, ill fit.

**Table 1:** Maximum likelihood estimates for the Browne et al [1] two-factor, and the progressive impact, models

	*Browne et al.[1] Model*^++^				*Progress ive Impact Model*						
	NK Activity Factor	IFN Activity Factor	Indicator R^2+++^		NK Activity Factor	IFN Activity Factor	NK Progressive Factor	IFN Progressive Factor	Indicator R^2^	Proportion of indicator variance explained by NK Activity Factor	Proportion of indicator variance explained by IFN Activity Factor
											
NK100	.842	.003	.709		.705**	--	-80.1**	--	.958	.50	--
NK50	.936	-.005	.876		.851**	--	-50.0+	--	.918	.72	--
NK25	.943	.015	.892		.920**	--	-25.0+	--	.874	.85	--
NK12	.964	-.013	.927		.922**	--	-12.5+	--	.995	.98	--
											
IFN50	.030	.942	.893		--	.897**	--	-50.0+	.972	--	.80
IFN25	-.019	.996	.988		--	.977**	--	-25.0+	.996	--	.95
IFN12	.005	.995	.990		--	.988**	--	-12.5+	.988	--	.98
IFN6	-.018	.991	.977		--	.944**	--	-6.25+	.990	--	.99
											
Factor Variance	1.0+	1.0+			1.0+	1.0+	.000069^§^**	.000069^§^**			
											

^+ ^a fixed coefficient

* beyond 2 standard errors

** beyond 3 standard errors

^§ ^constrained to be equal

^++ ^Identifying Browne et al's [1] exploratory two-factor model requires excluding one indicator from loading on each factor. Repeated emails to both Browne and MacCallum were unable to elicit a statement of precisely which two loadings had been set to zero. There are 16 different ways of excluding one IFN indicator from loading on the NK activity factor and simultaneously excluding one NK indicator from the INF activity factor (see dashed loadings in Figure 1). The loadings in column 1 and 2 are the average of the estimated loadings calculated across these 16 exclusion possibilities. Each of the 16 models provided a χ^2 ^= 103.8, df = 13, p = 0.000, with the slight difference from the reported fit being easily attributed to the three figure accuracy of the correlations published in Browne et al.[1].

^+++ ^These values are for the version of the Browne et al. [1] model that excludes the effects leading from the NK latent to IFN6 and from the IFN latent to NK100 for identification of the model.

Andersen, Farrar, Golden-Kreutz, Kutz, MacCallum, Courtney & Glaser [3] provide a description of the reasonably standard procedures used to obtain the Browne et al. [1] data. Peripheral blood leukocytes (PBLs) were obtained from 60 mL of venous blood, counted so that a known number of PBLs could be suspended in medium and incubated with either additional medium or additional medium plus rIFNγ. K562 target cells (a human myeloid cell line sensitive to NK cell activity) were labelled with ^51^Cr and aliquoted with the effector cells (either the NK, or the rIFNγ activated NK cells) in the ratios reported above. The cell mixture was centrifuged to ensure cell surface contact, and incubated to provide an opportunity for the NK cells to bind and rupture the target cells, thereby releasing the radioactive target cell cytoplasm. Gamma radioactivity of the supernatant collected from a second centrifuging indicated the effectiveness of the NK or rIFNγ-activated-NK cells at lysing the target cells, with larger measurements corresponding to more effective NK cell activity.

### An alternative model

Browne et al. [1] modelled the measurements made at the various E:T ratios as replicate measurements. Hayduk suspected that the progressively varying E:T ratios might have introduced systematic measurement interference. Higher E:T ratio measurements might result in systematically less NK cell effectiveness, not because of differential NK activity but because of some progressive complication subsumed within the measuring procedures. For example, higher E:T ratios might decrease the ability of NK cells to contact and lyse target cells due to competition for cell surface contact area. Or multiple NK (or other leukocyte) cells might block some NK cells from attaining sufficient surface contact with the K562 cells, and thereby render them seemingly ineffective – not due to lack of potency, but as a result of competition for surface contact. Alternatively, the lysing of a single target cell by multiple attached NK cells, which becomes more likely at higher E:T ratios, might make the NK cells appear comparatively ineffective on a "per cell" basis. The amount of target cell cytoplasm released per effector cell would be disproportionately small because multiple NK cells might have to "share the credit" for participating in lysing a single target cell, and not because of lower NK cell effectiveness. Competition for attachment sites, and multiple simultaneous NK attacks on single targets, would increase as the effector NK cells more radically outnumbered target cells, and hence should be more pronounced at higher E:T ratios.

**Figure 2:**
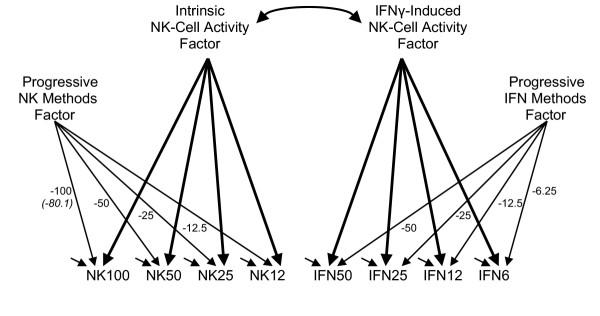
The model with progressively influential factors

These considerations led to the model of E:T-progressive interference depicted in Figure 2. This model postulates two latent factors, paralleling the factors in the Browne et al. [1] model (an NK activity factor causing the NK indicators' values, and an rIFNγ activity factor causing the IFN indicators' values), plus two progressively interfering factors (one spanning the NK indicators, the other spanning the IFN indicators). The effects of the interfering factors are progressive in proportion to the E:T cell ratios, and negative because we anticipated progressive reduction in the per-cell radioactivity readings, as discussed above. The negative signs are purely for ease of expression, since progressive positive values result in an equivalent model that merely interchanges the high and low ends of the underlying factor's scale. One progressive factor is postulated as acting within each measurement series, and these factors are postulated as being independent of one another, and also independent of the true scores on the NK and rIFNγ-enhanced activity factors. The variances of the two methods factors were constrained to be equal because the procedural similarity in the measurement series initially led us to suspect that routine within-series laboratory variations might propagate proportionally. We originally saw no reason to anticipate that rIFNγ would alter the mechanisms initially postulated as providing the progressive interference. Later consideration of multiple potential mechanisms led us to investigate the possibility of variance differences, as reported below.

## Results

**Figure 3:**
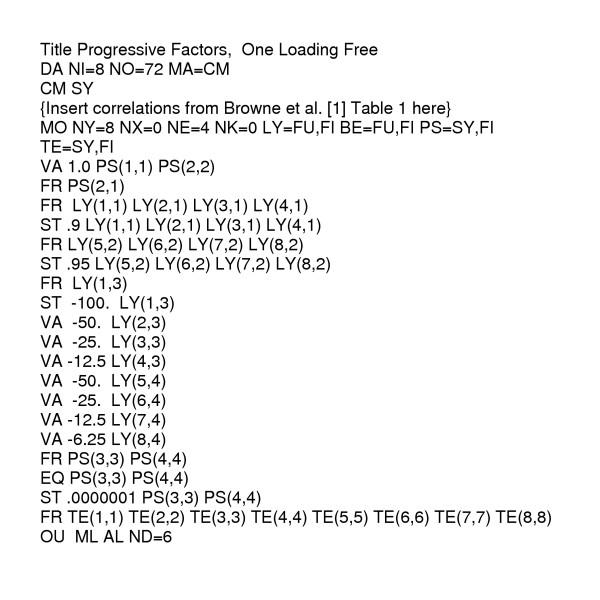
LISREL (Joreskog and Sorbom [6]) program syntax for the Figure 2 progressive factors model

This model contains 18 estimates: four loadings of the NK indicators on the NK activity factor, four loadings of the IFN indicators on the rIFNγ activity factor, the correlation between the two activity factors (whose variances are fixed at 1.0), eight measurement error variances (one per indicator), and the single variance applied or assigned to both the separate interfering factors. This model fits, but a negative measurement error variance estimate for NK100 suggested a ceiling had been reached for the largest E:T ratio. Freeing the loading for the NK100 indicator results in clean fit (χ^2 ^= 11.97, *df *= 17, *p *= 0.802) and an estimate of -80.1, rather than a strictly proportional value of -100. The alternative of constraining the offending measurement error variance to be non-negative while maintaining the -100.0 loading, also results in a fitting model (χ^2 ^= 14.72, *df *= 18, *p *= 0.681) having very similar estimates, so whether the interfering effects are "nearly proportional" or "strictly proportional" is equivocal. The progressive and nearly-proportional model (see Figure 3 for program details) provides the estimates in Table 1. The clean fit of this model convinces us that something is indeed interfering with the NK cell activity measurements, and "that something" is acting progressively and nearly in proportion to the E:T ratios.

### Further characterising the interfering entity

Additional models were estimated in attempts to further characterize the entity providing the progressive interference. The lowest inter-item correlations, and the greatest ill-fit of the Browne et al. [1] model, appeared for the NK measurements, so we checked whether a single methods factor spanning only the NK measures would be consistent with the data. This model is similar to Figure 2 model, except that the progressive methods factor spanning the IFN measures is eliminated. This model fails significantly (χ^2 ^= 62.5, *df *= 17, *p *< 0.001) and thereby informs us that even the seemingly cleaner IFN measurements are influenced by some progressively interfering factor.

Are two similar, yet separate, factors required for the NK and IFN measurement series, or might it be possible that one E:T-coordinated progressive factor spans all eight measurements? That is, could the progressive interfering factor be something common to an individual, rather than something set for each individual once within the NK measurement series and reset independently (or be some other interference) within the IFN measurement series? To check this, we specified a model having a single progressive methods factor leading to all eight indicators. The same E:T ratio dictated loadings were used, and the NK100 loading freed. This model also fails convincingly (χ^2 ^= 75.5, *df *= 17, *p *< 0.001) and thereby speaks against the progressive entity being something connected to each case as a whole. That is, no single feature common to the full set of measurements (e.g. the time of blood sampling, or the person's age, or their cancer progression, or mistaken cell counts), could be progressively applied across both measurement series to account for the data. Such factors might constitute the entity spanning the items within one series, but the other measurement series would have to be progressively influenced by something else.

Together, these two failing models require that the entities providing the progressive interference are features connected exclusively to either the NK series or the IFN measurement series, or are something that is set independently within each of the NK and IFN measurement series for any given case.

We next attempted to check the requirement for equal progressive-factor variance incorporated in the Figure 2 model. Attempting to estimate a separate variance for each progressive factor resulted in signs of under-identification, and hence these data should be heard as being consistent with, but not necessarily as requiring, equal variances for the progressively interfering factors.

In response to the comments of Reviewer-2 (Professor Mulaik), we attempted to check whether the progressive methods factors were necessarily independent of the corresponding true NK activity factors, by freeing the corresponding covariances (which were constrained to be equal). This also resulted in signs of underidentification. Hence these data should be heard as being consistent with, but not necessarily as demanding, the independence of the methods factors from the corresponding true NK activity factors. Forcing even a modest correlation between true NK activity and progressive methods factors results in substantial suppression of effects, and standardized effects exceeding 1.0 – which is not "impossible" but which would certainly "confront" anyone inclined to postulate a coordination between true NK activity and the progressive methods factors on substantive rather than "exploratory-statistical" grounds.

It might be tempting to interpret these underidentified models as signs of insufficient power due to the rather small N of 72 provided by Browne et al. [1] but we think it would be more reasonable to see these underidentified models as artifacts of the limited variety of variables in the Browne et al. [1] data. The N of 72 provided sufficient power to speak strongly against the Browne et al. model, and sufficient power to speak strongly against several of the alternative models we considered above. The models that became underidentified did so largely because the structure of these models resulted in the freed coefficients having no unique (freed-coefficient dependent) implications which could potentially be found to be more/ less consistent with the data. Anyone wishing to investigate the ideas contained in the underidentified models would be better advised to add a wider variety of variables into their data and model structure, rather than merely increasing N while maintaining the current style of measurements and models.

### The parameter estimates

We have basically two sets of estimates to consider: the estimates for the failing Browne et al. [1] two-factor model (Figure 1, and Table 1 columns 1, 2, and 3), and the estimates for the fitting progressive measurement interference model (Figure 2, and Table 1 columns 4 through 10). The loadings of the measures on the NK-activity and IFN-activity factors, namely the estimated effects of "true" NK-activity and "true" IFN-activity on their respective sets of four measures, differ importantly between these models. The Browne et al. [1] estimates are relatively uniform and large, in contrast to the loadings for our fitting model which display a definite progression from smaller to larger loadings as one moves from higher to lower E:T ratio measurements.

It is no coincidence that the weakest loading estimates appear where the progressive interference is the greatest, namely for the highest E:T ratio measurements. As more variance in a measure is accounted for by the progressively interfering factor, less variance is left to be accounted for by the true NK or IFN activity factor. According to the fitting progressive model, only about half the variance in the NK100 indicator arises from true variability in NK activity, while the "other half" of the variance arrives primarily from the progressive methodological factor, with a minimal amount of error variance contributed by features unique to the NK100 measurement.

Given that the latent variables have variance 1.0, the variance the NK activity factor contributes to an NK indicator can be calculated as the square of the appropriate loading. The Browne et al. [1] model, therefore, claims true NK activity contributes .71, .88, .89 and .93 to the variance in NK100, NK50, NK25 and NK12 respectively. In contrast, squaring the effects leading from our NK activity factor to the indicators provides values of .50, .72, .85 and .98. These values make it clear that Browne et al.'s [1] overlooking of the progressive methodological interference results in their model claiming that too large a portion of the variance in the high E:T ratio measures arises from true NK activity, while too small a portion of the variance in the lowest E:T ratio measure arises from true NK activity. (A similar, but less pronounced, pattern appears if corresponding calculations are made for the contribution of IFN activity to the IFN indicators.) That is, the bias in the Browne et al. [1] estimates systematically obscures the substantial and progressively stronger measurement of true NK activity by the lower E:T ratio measurements, whether viewed from the perspective of the estimates themselves or the variance accounted for by those estimates.

The squared multiple correlation coefficient R^2 ^(column 3 of Table 1) is usually interpreted as a "proportion of explained variance" but the above observations require that we reconsider this for the Browne et al. model. The Browne et al. model fails to fit with the data, and hence confronts evidence of causal misspecification, and it also confronts evidence of bias in its estimates. Is it reasonable to claim that a misspecified model containing biased estimates "explains" or "accounts for" variance in the indicators? Even biased estimates can be put through the mathematical formula providing model-implied variances and R^2 ^(see Hayduk [5] pages 106–116, 184; and notice that the first four entries in column 3 correspond closely to the model-implied variance contributions reported in the preceding paragraph), but can mathematically-clean manipulations of biased, non-world-matching, coefficients be reasonably described as providing an "account of" or an "explanation for" indicator variances? That is, if biased estimates from a wrongly specified model are put through the perfectly-adequate mathematics providing variance implications, are the resultant variances "explained" or "accounted for"?

Our view is that claims to "explaining variance" and "accounting-for variance" are rendered unconvincing if there is evidence indicating the model that supposedly provides the "explanation or account" fails to correspond to a proper representation of the external world. Hence, we view the R^2 ^values in column 3 of Table 1 as properly calculated, yet fundamentally dubious, because the calculations are based on biased estimates from a misspecified model. These R^2 ^values constitute "dubiously explained or accounted-for proportions" of indicator variances.

Our Figure 2 model does not confront evidence of misspecification, and hence it would seem that the R^2 ^values in column 8 of Table 1 could be more comfortably described as proportions of explained variance. But these R^2 ^values have a different kind of uncertainty attached to them because the identity of the progressive latent variable is currently unascertained, as we discuss in the next section. The final two columns of Table 1 provide the proportions of variance in the indicators that are most confidently "explained" because these values come from a model that fits the data, and report the proportions of variance originating in latent variables whose identity is most confidently known.

Let us next consider the loading estimates from the perspective of the correlations between two pairs of indicators, specifically the correlation between the NK100 and NK50 measurements (0.902) and the correlation between NK25 and NK12 (0.930). The Figure 2 model accounts for the 0.902 NK100-NK50 correlation via the action of two common causes: the true NK activity factor which contributes (0.705)(0.851)(1.0) (namely, the product of two loadings and the variance of the relevant common factor; Hayduk [5] pages 26, 106), and the progressively interfering factor which contributes (-80.1)(-50.)(0.0000695), for an overall correlation of 0.600 + 0.278 = 0.878 (with the remaining 0.023 residual being within the range of sampling fluctuations). The 0.930 NK25-NK12 correlation is similarly accounted for by a true NK activity contribution (0.920)(0.992)(1.0) and a progressive methods factor contribution (-25.)(-12.5)(0.0000695), for a total of 0.912 + 0.022 = 0.934 (which leaves a residual of -0.004).

Notice that while the correlations are not radically different (0.902 vs 0.930) the contribution to the correlation provided by the causal actions of true NK activity differ substantially (0.600 versus 0.912). A substantial portion of the correlation between the NK100 and NK50 measurements is being provided by the progressively interfering factor, and when this is taken into account, there is a substantial reduction in the degree of coordination that can be attributed to both these measures causally responding to true NK activity. This is the classic distinction between reliability and validity. The NK100 and NK50 measures seem to possess substantial reliability (the basic 0.902 correlation) but much less validity since a substantial portion of the stability, or inter-measure reliability, is arising from a stable, and in this instance progressively-influential interfering entity.

The small variance estimate for the progressively interfering factor (0.0000695) is partially an artifact of the large absolute values used in setting the proportional methods effects (-100, -50, -25, etc.). If each of these effects is rescaled by dividing by 100, the effects become -1.0, -0.5, -0.25, -0.125 for NK and -0.5, -0.25, -0.125 and -0.0625 for IFN, and the proportionality of the effects is preserved but the estimated variance of the progressive factor is increased 100^2 ^fold, to 0.695, while the other estimates remain unchanged.

One additional model estimate is worth noting. The Figure 2 model permits a correlation between the NK and IFN activity factors, but the corresponding estimate is small (0.090) and insignificant. The insignificance of this correlation implies that it is reasonable to view all four of the factors in Figure 2 as being basically independent of one another. Two independent entities account for the NK measurements while two additional entities that are independent of one another and also independent of the NK-measurement-producing entities account for the IFN measurements.

### What is producing the progressive interference?

Let us first consider features capable of producing progressive interference within each series. Multiple simultaneous lysings of a single target cell provide several possibilities. With higher E:T ratios it becomes progressively more likely that any given target cell will be simultaneously attacked by more than one NK cell. The ^51^Cr "credit" for having lysed a target cell will be shared among the multiple attacking NK cells, and hence will reduce the seeming per-NK-cell effectiveness of the NK cells. Individual differences in the mechanisms of cell recognition, strength of attachment, delay in NK cytoplasmic reorganization, or energy supply, which are separate from whatever rate-limit constitutes "true NK cell activity", could provide individual differences constituting the variance in the "progressive factor". From this perspective, the independent progressive factor within the rIFNγ series might constitute a rIFNγ induced switch to a different rate-limiting component associated with multiple NK lethal attachments.

Alternatively, the progressive interference might arise from the blocking of some effector NK cells by physical presence of scrimmage-line NK or lymphocyte cells. If an NK cell is obstructed or delayed in making contact with a target cell by: a) the physical obstruction created by other cells between this NK cell and the target, or b) the NK cell wasting time discovering that the adhered cell is merely another NK or lymphocyte cell rather than a valid target, this progressively reduces the apparent ^51^Cr-producing effectiveness of that cell – again a phenomena which should coordinate with the E:T ratio.

The "multiple simultaneous attacks" and "blocking" scenarios might be supplemented by individual differences in the ability of NK cells to lyse multiple sequential targets. At higher E:T ratios fewer pristine targets are available and hence fewer NK cells have the opportunity to deliver second-lethal-doses, or may end up sharing their second-dose credits. Or, if lethal doses from multiple NK cells reduce the time to ion-gradient-induced cell membrane rupture, multiple-simultaneous-NK activity might instantiate the positive-valued model reported above.

Yet other possibilities arise from NKT-cells and T-cell suppression of NK cells. At higher E:T ratios, there may be greater suppression of NK cells by higher concentrations of suppressor chemical signals. Similarly, NKT-cells may become progressively activated or deactivated by E:T-concentration-dependent signals from other T cells in the medium.

If an individual's NK cells are not uniformly active, but rather display a within-individual gradient of activity (some NK cells being more active than others), and if this gradient is set independently of the features underlying "true NK activity" this would provide another form of explanation. Yet another possibility arises from the uptake of ^51^Cr by NK cells or other lymphocytes following its release from lysed target cells. At higher E:T ratios more cells are present to re-uptake ^51^Cr released from lysed target cells, and hence less ^51^Cr will appear in the centrifuged supernatant. Clearly, there are multiple possibilities for what might be providing the E:T ratio coordinated variations in lysing ability, and any independent pairing of these possibilities potentially constitute the interference in the NK and IFN measurement series.

## Discussion and Conclusions

This study was prompted by fortuitous use of NK cell activity measurements in a debate over the testing of structural equation models. According to Browne et al. [1], even though the two-factor model of NK and rIFNγ activity they proposed (Figure 1) was significantly at odds with their correlation data, the residual differences were small enough to be "negligible from a practical point of view". Our view was that the small size of the correlation residuals did not imply that the reason for the ill fit was correspondingly small or unimportant, and this prompted our reexamination of what might be producing the ill fit. These reconsiderations led to the Figure 2 model in which the measurements reflect both the "true" degree of NK or rIFNγ induced NK cell activity along with the influences of features that progressively impact these measurements in proportion to the E:T ratios. Introducing a progressive, and nearly proportional, interfering factor within each measurement series resulted in a cleanly fitting model whose residuals are small enough to be easily attributable to chance sampling fluctuations, and whose estimates imply that true NK or rIFNγ-induced activity is most accurately measured at low E:T ratios. The impact of the progressively interfering feature is sufficiently pronounced that at the highest E:T ratio of 100 only half of the variance in the NK measurement can be attributed to "true" NK activity. The "other half" of the variance in this measurement seems to arise primarily from the progressive factor. Thus the small residuals of the Browne et al. [1] model seem to have obscured major influences in the data. Consideration of the methodology underlying the NK and rIFNγ measurements locates several possible identities for the progressively effective feature, including multiple simultaneous lysings, cell occlusion or blockage, within-individual NK activity gradients, and ^51^Cr reuptake.

One important consequence of the fitting model is that it provides evidence indicating the lowest E:T measures provide the most valid measures of NK and rIFNγ induced lysing activity. The NK or rIFNγ measurements made at higher E:T ratios correlate highly with one another, but a substantial portion of these correlations appears to result from the progressive interfering factor and not "true" NK and rIFNγ activity. The higher E:T ratio measures remain reliable in the sense of being stable, but they are not as valid as measurements of "true" lysing activity. Given that nearly half the variance in the NK100 measurements is connected to the progressive factor, we have encountered something that is substantial and probably routinely noticed in practice, and is just being mislabeled or overlooked.

A second important consequence of the Figure 2 model is that it suggests that there may exist some "third-causal-source" of lysing ability. If we think of natural lysing ability as a first source, and rIFNγ activation as a second source of lysing ability, the progressive factor may constitute a third and independent causal source. That is, just as rIFNγ-induced NK cell activity can therapeutically supplement NK activity, whatever constitutes the progressive factor may also be able to therapeutically supplement both the NK and rIFNγ-induced activities. If the interfering factors turn out to be something like blocking of access to the target cells by other cell bodies, this will be viewed as merely "the reason" lower E:T ratios provide more trustworthy measurements. But if the interfering factor turns out to be something connected to a chemical concentration (e.g. magnesium stores) then this could constitute a potential third and independent causal route to therapeutic enhancement of killer cell activity.

The fact that the progressive factors are tightly connected to E:T ratios makes differential NK activity at various E:T ratios an obvious point of investigative departure. The fact that one of the progressive factors contributes about half the variance in the highest E:T ratio NK measurements implies we are not confronting issues at the limits of measurement, but rather are confronting issues of measurement confounding. Incorporating measures of variables connected to the "candidate explanations" in an expanded version of Figure 2 could effectively screen the explanatory options.

## Competing interests

The author(s) declare that they have no competing interests.

## Authors' contributions

LAH developed and ran the structural equation models, and prepared multiple drafts of the manuscript. HPR discussed, and suggested changes to, the various manuscript drafts; prepared the figures, and arranged for consultation with outside experts. GGC discussed, and suggested changes to, the various drafts; and arranged for consultation with outside experts. MJDL discussed, and suggested changes to, the various drafts; and prepared the table. MAB discussed, and suggested changes to, the nearly final manuscript drafts. All the authors read and approved the final version of the manuscript.

## Pre-publication history

The pre-publication history for this paper can be accessed here:


